# Analysis of the 60/60 Sign and Other Right Ventricular Parameters by 2D Transthoracic Echocardiography as Adjuncts to Diagnosis of Acute Pulmonary Embolism

**DOI:** 10.7759/cureus.13800

**Published:** 2021-03-10

**Authors:** Bhupesh R Shah, Subrahmanya Murti Velamakanni, Aman Patel, Gajanan Khadkikar, Tejas M Patel, Sanjay C Shah

**Affiliations:** 1 Cardiology, Smt NHL Municipal Medical College, Ahmedabad, IND; 2 Interventional Cardiology, Apex Heart Institute, Ahmedabad, IND

**Keywords:** pulmonary embolism, echocardiography, 60/60 sign

## Abstract

Introduction

The 60/60 sign in 2D transthoracic echocardiography (TTE) - a combination of pulmonary acceleration time (PAT) less than 60 milliseconds and tricuspid regurgitation (TR) jet gradient of less than 60 mmHg - has been found to be specific for the diagnosis of pulmonary embolism (PE).

Materials and methods

An observational prospective analysis was carried out on cases of suspected PE presenting to the emergency room (ER). TTE was performed on all cases with suspected PE prior to computed tomography pulmonary angiography (CTPA). Emphasis was placed on measurement of PAT and early systolic notching (ESN) on the pulsed wave (PW) Doppler of the pulmonary valve, TR jet gradient, right ventricle systolic excursion velocity (RV S’) by tissue doppler imaging (TDI), tricuspid annular plane systolic excursion (TAPSE), and right ventricle to left ventricle end-diastolic dimension ratio (RV:LV EDD) in modified parasternal short-axis view. These signs were taken as screening tests and compared to CTPA as the standard test. Patients were followed up until hospital discharge or death.

Observations

Fifty-six cases of suspected PE were enrolled for the study. Of these, 24 cases of PE were confirmed by CTPA. Out of 24 cases of PE, 15 were high-risk PE, six were intermediate high-risk PE, and three were intermediate low-risk PE. The mean age was 53.07±9.79 years with a male-to-female ratio of 1.95:1. The 60/60 sign was present in 70.83% of cases of PE. RV:LV EDD in a modified short-axis view of more than 0.9 was present in 91.67% of cases of PE, and ESN on the PW Doppler of the pulmonary valve was present in 75% of cases of PE. The 60/60 sign, RV:LV EDD ratio more than 0.9, and ESN showed sensitivities of 70.83%, 91.67%, 75%, and specificities of 93.75%, 75%, and 100%, respectively for PE. For prediction of mortality, presence of the 60/60 sign (Odds Ratio=8.13, p-value=0.034) and ESN (Odds Ratio=17.50, p-value=0.02) were statistically significant.

Conclusions

60/60 sign and ESN are specific for the diagnosis of PE but have poor sensitivity.

## Introduction

The current standard of diagnosis of acute pulmonary embolism (PE) is computed tomography pulmonary angiography (CTPA). There has been sustained interest in the role of transthoracic echocardiography (TTE) as an aid for the diagnosis of PE. The advantage of TTE is in it being available as a rapid point-of-care tool. In many situations, particularly in high-risk PE with hemodynamic instability, a CTPA may not be feasible immediately. The latest European Society of Cardiology (ESC) guidelines for PE in 2019 mandate the use of TTE as a first-line investigation in patients of high-risk PE with hemodynamic instability. Further, in the same subset of patients, if CTPA is not feasible, then a treatment based on clinical and TTE findings is likely justified [1]. In this background, the 60/60 sign - a combination of tricuspid regurgitation (TR) jet gradient less than 60 mmHg and a pulmonary valve acceleration time (PAT) less than 60 milliseconds (ms) - has been studied as a marker of acute right ventricular (RV) strain. The 60/60 sign has been found to highly specific for the diagnosis of PE, albeit at the cost of poor sensitivity [2]. In the same context, other right ventricular parameters, including RV dilation, RV dysfunction, McConnell’s sign, and RV outflow Doppler changes have also been studied in PE [3-4]. In this background, a prospective study was undertaken to evaluate the accuracy of these echocardiographic signs as compared to the gold standard test of CTPA in patients of suspected PE presenting to the emergency room.

## Materials and methods

A hospital-based prospective analysis was done on all patients admitted to the emergency room with suspected acute pulmonary embolism over a period of 10 months in 2019. Patients with sudden onset of breathlessness, chest pain, or syncope were first screened in the emergency room. All these patients underwent basic bedside physical examination, electrocardiography, bedside TTE, and x-ray of the chest where appropriate to rule out other major causes of an acute presentation, namely - acute coronary syndromes, acute decompensated heart failure, severe acute chronic obstructive pulmonary disease, aortic dissection, etc. After these conditions were ruled out, patients were classified as suspects for acute PE and evaluated accordingly.

**Figure 1:**
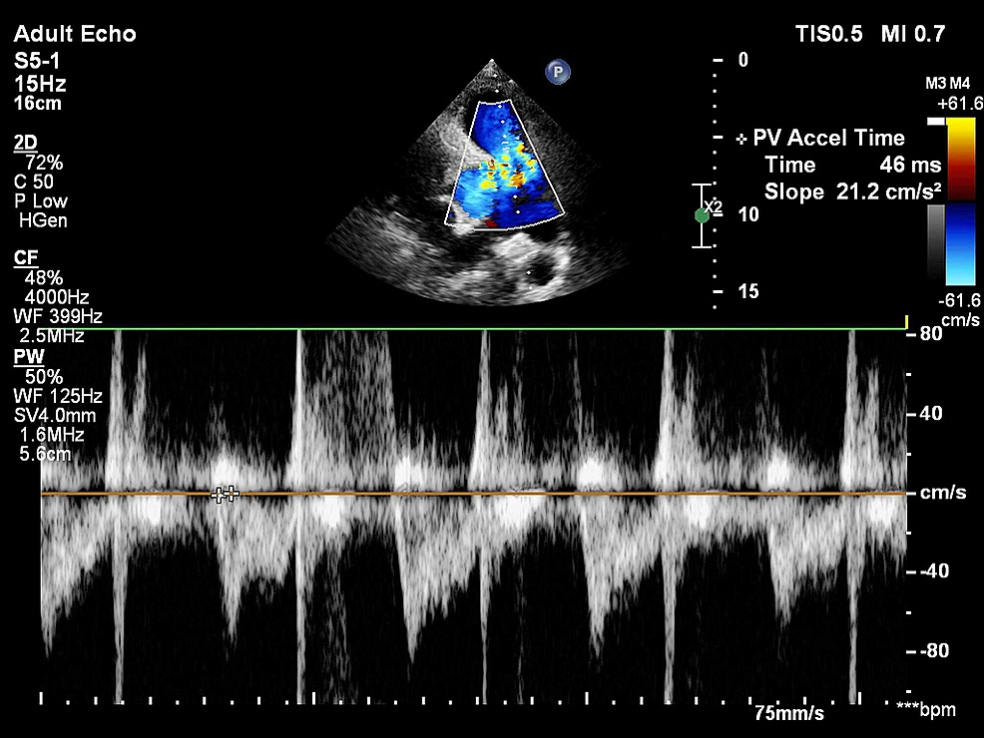
Pulsed wave (PW) Doppler showing a reduced pulmonary acceleration time (PAT) and early systolic notching (ESN)

**Figure 2:**
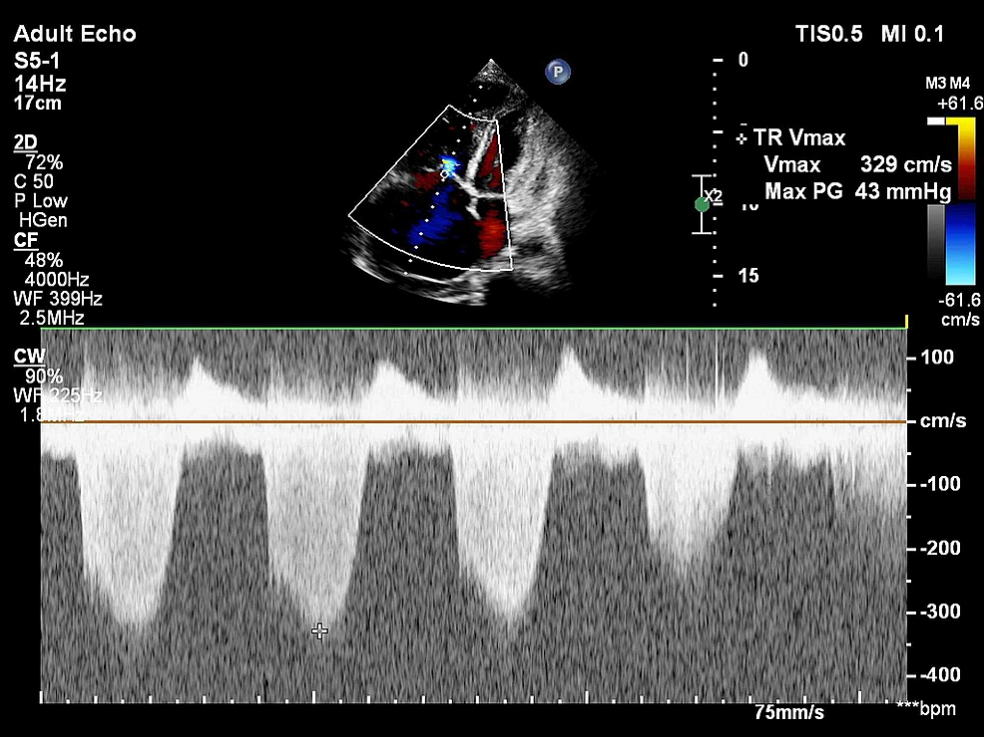
Continuous wave (CW) Doppler signal of the tricuspid valve regurgitation jet

**Figure 3:**
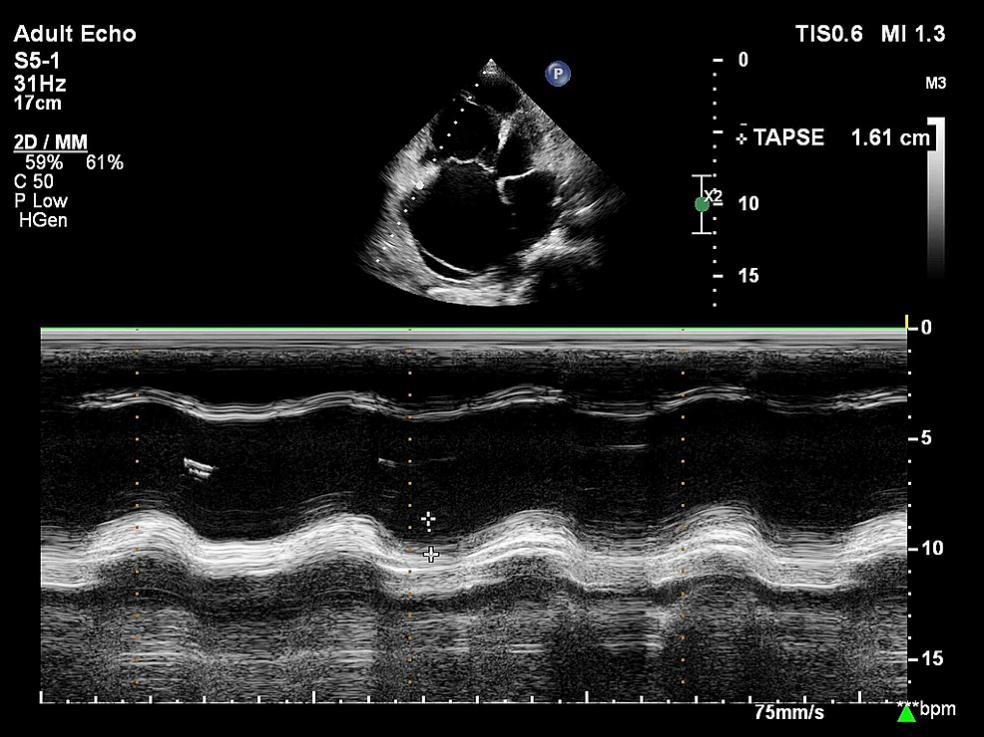
Motion mode echocardiography section demonstrating tricuspid annular plane systolic excursion (TAPSE)

**Figure 4:**
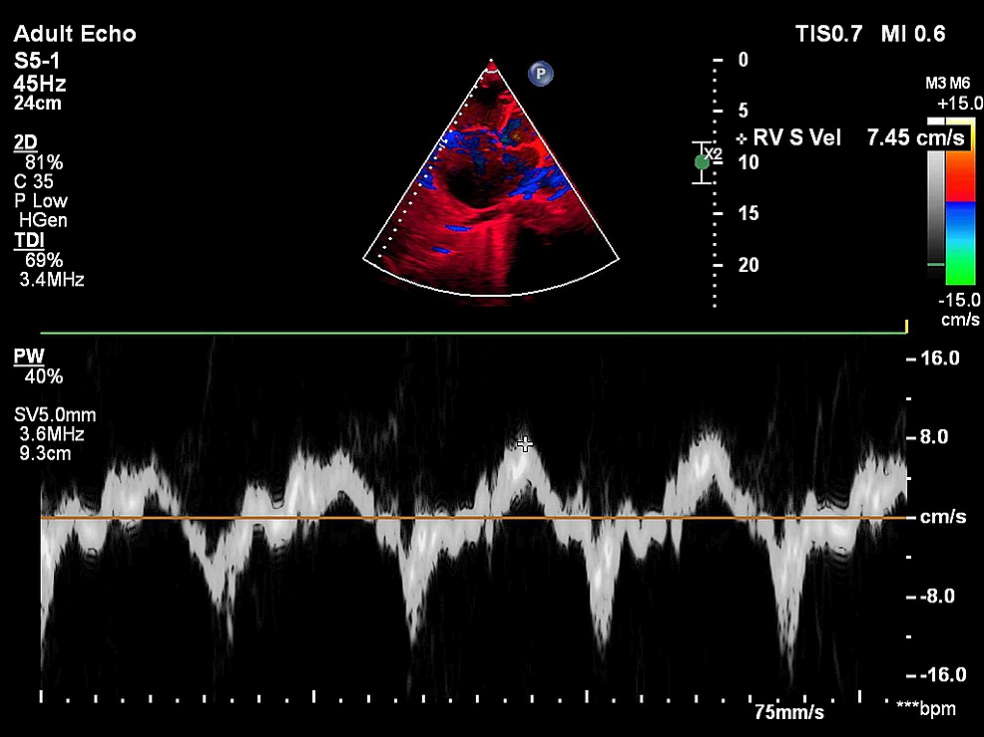
Tissue Doppler imaging showing right ventricle systolic excursion (RV S') velocity

All patients suspected of acute PE underwent bedside TTE using the EPIC-7C cardiovascular imaging system (Koninklijke Philips NV, Amsterdam, the Netherlands). Emphasis was placed on the evaluation of the following right ventricular parameters - pulmonary acceleration time (PAT) and presence of early systolic notching (ESN) on the pulsed-wave (PW) Doppler signal of the pulmonary valve in short-axis view (Figure 1); estimation of TR jet velocity maximum gradient (Figure 2); tricuspid annular plane systolic excursion (TAPSE) (Figure 3) and RV systolic excursion velocity (RV S’) velocity by tissue Doppler imaging (TDI) in the apical four-chamber view (Figure 4); right ventricle to left ventricle end-diastolic dimension ratio (RV:LV EDD) in a modified short-axis view (Video 1). The short axis view must be obtained at the left ventricular basal level where the mitral chordae are visible. The 60/60 sign was considered present when the TR jet gradient was less than 60 mmHg along with a pulmonary acceleration time of less than 60 milliseconds (Figure 1). An RV:LV EDD ratio of more than 0.9 was taken as a cutoff for RV dilation. TAPSE less than 17 millimeters (mm) and an RV S’ velocity less than 10 cm/s were considered evidence of right ventricular dysfunction. These cut-off reference values are taken from the American Society of Echocardiography (ASE) guidelines [5].

**Video 1:** Modified parasternal short-axis view showing the optimal view required for calculating right ventricle to left ventricular internal dimension ratio in end-diastole

All patients were then risk stratified. Risk stratification was done using the ESC guidelines [1]. If a patient had hemodynamic instability (defined as a systolic blood pressure ≤90 mmHg), a simplified pulmonary embolism severity index (sPESI) score ≥1, RV dysfunction on TTE, and elevated cardiac troponin levels, the patient was classified as high-risk PE. If a patient had RV dysfunction on TTE, sPESI score ≥1, and elevated cardiac troponins with no hemodynamic instability, the patient was classified as intermediate high-risk PE. If a patient had an sPESI score ≥1 along with either RV dysfunction on TTE or elevated cardiac troponins only, the patient was classified as an intermediate low-risk PE. The sPESI score assigns one point score each to - age more than eighty years, history of cancer, history of chronic cardiopulmonary disease, pulse rate ≥110 beats per minute, systolic blood pressure less than 100 mmHg, and an arterial blood oxygen saturation less than 90% [6].

Patients then underwent CTPA using the Ingenuity core 128 slice machine (Koninklijke Philips NV, Amsterdam, the Netherlands) using a PE protocol with iodinated contrast at 3-4 ml/second with an iodine concentration of 370 mg/ml. CTPA was done immediately when feasible, or after immediate resuscitation and thrombolysis in case of high-risk PE. The presence of contrast filling defects on the CTPA was taken as definite positivity of PE and used as a gold standard test for comparison. Patients were then followed up until hospital discharge or death.

Statistical analysis

Statistical analysis was carried out using Statistical Package for the Social Sciences (SPSS), version 23.0 (IBM Corp, Armonk, NY, USA). Echocardiographic parameters were considered as screening tests and compared to the gold standard test, CTPA.

## Results

A total of 56 cases of suspected PE were enrolled for the study. Of these, 24 cases of PE were confirmed by CTPA and the rest did not have any thrombi on CTPA. Out of 24 cases of PE, 15 were high-risk PE, six were intermediate high-risk PE, and three were intermediate low-risk PE. The mean age was 53.07±9.79 years with a male-to-female ratio of 1.95:1.

The 60/60 sign was present in 13 cases of high-risk PE, two cases of intermediate-high risk PE, two cases of intermediate low-risk PE, and two cases without PE. Overall, 60/60 sign was present in 70.83% of cases of PE. RV:LV EDD in a modified short-axis view of more than 0.9 was present in 91.67% of cases of PE, and ESN on the PW Doppler of the pulmonary valve was present in 75% of cases of PE (Table 1).

**Table 1:** Breakup of cases with positive echocardiographic signs PE - pulmonary embolism, RV:LV EDD ratio - right ventricle to left ventricle end-diastolic dimension ratio, ESN - early systolic notching of the pulsed wave Doppler signal of the pulmonary valve

Parameter	Number of cases with positive parameter				
	High-risk PE (n=15)	Intermediate High-risk PE (n=6)	Intermediate Low-risk PE (n=3)	Total cases of PE (n=24)	Without PE (n=32)
60/60 sign	13	2	2	17 (70.83%)	2 (6.25%)
RV:LV EDD Ratio > 0.9	15	6	1	22 (91.67%)	8 (25.00%)
ESN	13	4	1	18 (75.00%)	0

The 60/60 sign showed a sensitivity of 70.83% and a specificity of 93.75% for PE diagnosis as compared to the gold standard of CTPA. The RV:LV EDD ratio >0.9 showed a sensitivity of 91.67% and specificity of 75% as compared to the gold standard of CTPA. ESN showed a sensitivity of 75% and specificity of 100% for PE diagnosis (Table 2).

**Table 2:** Showing the sensitivity and specificity of various echocardiographic parameters as compared to the standard test of computed tomography pulmonary angiography CI - confidence interval, RV:LV EDD ratio - right ventricle to left ventricle end-diastolic dimension ratio, ESN - early systolic notching of the pulsed wave Doppler signal of the pulmonary valve

Parameter	Sensitivity (95% CI)	Specificity (95% CI)	Positive likelihood ratio (95% CI)	Negative likelihood ratio (95% CI)	Positive predictive value (95% CI)	Negative predictive value (95% CI)
60/60 sign	70.83% (48.91% - 87.38%)	93.75% (79.19% - 99.23%)	11.33 (2.89 - 44.43)	0.31 (0.17 - 0.58)	89.47% (68.43% - 97.09%)	81.08% (69.54%-88.95%
RV:LV EDD Ratio > 0.9	91.67% (73.00% - 98.97%	75.00% (56.60% - 88.54%	3.67 (1.99 - 6.76	0.11 (0.03 - 0.43)	73.33% (59.86% - 83.53%)	92.31% (75.82% - 97.87%)
ESN	75.00% (53.29% - 90.23%	100.00% (89.11% - 100.00%)	-	0.25 (0.13 - 0.50)	100%	84.21% (72.73% - 91.43%)

A total of 15 patients of PE out of 24 expired. On calculations of the above three parameters for prediction of mortality, the presence of the 60/60 sign (Odds Ratio=8.13, p-value=0.034) and ESN (Odds Ratio=17.50, p value=0.02) were statistically significant (Table 3).

**Table 3:** Odds ratios of prediction of mortality by various echocardiographic parameters CI - confidence interval, RV:LV EDD ratio - right ventricle to left ventricle end-diastolic dimension ratio, ESN - early systolic notching of the pulsed wave Doppler signal of the pulmonary valve

Parameter	Odds Ratio for predicting mortality	95% CI	P-value
60/60 sign	8.13	1.11 – 59.21	0.034
ESN	17.50	1.56 – 196.33	0.02
RV:LV Ratio >0.9	10.33	0.44 – 243.34	0.147

## Discussion

PE is an emergency requiring rapid diagnostic evaluation and treatment. In many cases, particularly high-risk PE, a CTPA may not be immediately feasible. This has prompted interest in the evaluation of parameters by TTE for PE diagnosis. The presence of an appropriate history, RV dysfunction and dilation by TTE in the setting of high-risk PE, when CTPA is not feasible, is adequate justification for treatment as per ESC guidelines [1]. The first well-described sign on a TTE is the McConnell’s sign. First described by McConnell et al. in a cohort of 41 patients, the sign describes a typical RV free-wall hypokinesis with apical sparing. The original study found the sign to be 94% specific and 77% sensitive [7]. A review of 199 patients found the McConnell’s sign to be poorly sensitive (21.74%) with good specificity (98.05%) [8].

The 60/60 sign was first studied in a cohort of 100 PE suspect patients by Kurzyna et al. [2]. The 60/60 sign was developed as it was thought to be less operator-dependent than the McConnell’s sign. The study found the sign to be specific, however, insensitive, for PE diagnosis. The largest evaluation of the 60/60 sign was done in an analysis of 511 consecutive PE patients by Kurnicka et al. [9] which found the sign to be present in 12.9% of patients. This is in contrast to the present study wherein 60/60 sign was seen in 70.83% of patients. The reason for this difference could be that the present study has a small sample, whereas the work of Kurnicka et al. was a large retrospective post-hoc analysis. A retrospective analysis of 67 patients suspected of PE found similar results of poor sensitivity for the 60/60 sign. The same study also found that the RV:LV EDD ratio was the most accurate for the diagnosis of PE [10]. These results match with the present study.

Early systolic notching (ESN) in the PW Doppler signal of the pulmonary valve has been evaluated by Afonso et al. in an analysis of 277 patients. The study concluded that the area under the receiver operator characteristic curve (AUC) of ESN was superior to the McConnell’s sign [4]. The present study also has a similar finding of the 60/60 sign and ESN being highly specific and the RV:LV EDD ratio being the most sensitive for the diagnosis of PE. All the above evidence demonstrates the fact that TTE is an important tool for the diagnosis of PE, but the standard remains CTPA. All the described TTE signs of PE are poorly sensitive but are relatively specific. In the presence of a high pre-test probability of PE, the signs may serve as important clues for the diagnosis of PE.

Study limitations

The sample size is small, which makes it difficult to draw definitive conclusions. Patients of low-risk PE were not included in the study.

## Conclusions

60/60 sign and early systolic notching (ESN) of the pulsed wave (PW) doppler of the pulmonary valve are specific for the diagnosis of PE but have poor sensitivity. Although TTE is the first-line investigation for a patient of suspected high-risk PE with hemodynamic instability, TTE remains only an adjunctive tool for PE diagnosis with the standard being CTPA. In the presence of a high pre-test probability, TTE signs may be helpful to make clinical decisions for treatment, particularly when CTPA may not be immediately feasible.
